# Generation and Analysis of Large-Scale Data-Driven *Mycobacterium tuberculosis* Functional Networks for Drug Target Identification

**DOI:** 10.1155/2011/801478

**Published:** 2011-11-29

**Authors:** Gaston K. Mazandu, Nicola J. Mulder

**Affiliations:** Computational Biology Group, Department of Clinical Laboratory Sciences, Institute of Infectious Disease and Molecular Medicine, University of Cape Town, Rondebosch 7701, South Africa

## Abstract

Technological developments in large-scale biological experiments, coupled with bioinformatics tools, have opened the doors to computational approaches for the global analysis of whole genomes. This has provided the opportunity to look at genes within their context in the cell. The integration of vast amounts of data generated by these technologies provides a strategy for identifying potential drug targets within microbial pathogens, the causative agents of infectious diseases. As proteins are druggable targets, functional interaction networks between proteins are used to identify proteins essential to the survival, growth, and virulence of these microbial pathogens. Here we have integrated functional genomics data to generate functional interaction networks between *Mycobacterium tuberculosis* proteins and carried out computational analyses to dissect the functional interaction network produced for identifying drug targets using network topological properties. This study has provided the opportunity to expand the range of potential drug targets and to move towards optimal target-based strategies.

## 1. Introduction

Throughout history, infectious diseases caused by microbial pathogens have had a devastating impact on human morbidity and mortality, and they remain of great concern, even today. With the advance of new high throughput sequencing technologies, there has been an increase in the number of worldwide microbial genome sequencing projects (http://microbialgenome.org, http://www.ncbi.nlm.nih.gov/genomes/lproks.cgi?view=1, http://www.sanger.ac.uk/Projects/Microbes and http://www.tigr.org/tdb/mdbcomplete.html), which has yielded complete genome sequences of crucial microbial pathogens of humans, animals, and plants. Analyses of these genome sequences have provided valuable insights into the dynamics driving pathogenic mechanisms and numerous virulence factors and have shed light on the targeted organism's biology [1]. The characteristic features of pathogenic organisms include their ability to colonize a specific host organ or tissue, to adapt to their environment, and to evade the host immune response [2], thus leading to the development of disease, as a result of a delicate and dynamic balance between pathogen and host defence system.

Furthermore, the availability of these pathogenic microbial genomes can contribute to speeding up the process of drug target selection [3] by finding genes that are essential to microbial cell survival or growth and virulence. In fact, significant progress has been made in drug discovery and vaccine administration against major infectious diseases [4]. However, these efforts are weakened by an increased incidence of widespread drug-resistant strains to the available and commonly used antibiotics and vaccines, a growing prevalence of infections, and the emergence of new pathogenic organisms, making infectious diseases the leading cause of human death worldwide. Tuberculosis (TB) is the biggest component of these infectious diseases, which claimed 1.8 million victims in 2008, and there were estimates of 9.4 million new cases that year (3.6 million of whom are women), including 1.4 million cases among people living with Human Immunodeficiency Virus (HIV) or Acquired Immunodeficiency Syndrome (AIDS) according to the World Health Organization (WHO) [5, 6].

TB is caused by an intracellular pathogen *Mycobacterium tuberculosis* (MTB), also known as tubercle or Koch's bacillus, whose genome sequence has been completely elucidated [7–9]. The complete elucidation and publication in 1999 [8] of the first MTB genome sequence constitutes the biggest step towards understanding MTB virulence and its specific abilities for invasion and division inside host macrophages. This has facilitated the identification and function prediction of all MTB proteins and the identification of genes common to all bacteria or specific to MTB. Even though there are still a large number of uncharacterized genes, which limits genomic studies, such data has provided a basis for selecting potential drug targets from the complete list of proteins. The genes in the MTB genome, but missing from closely related genomes, are likely to be crucial to its pathogenicity and constitute promising candidates for drug targets [3]. This shows that the use of available data and computational methods may help us better understand the mechanisms of virulence of MTB and features that enable this organism to adapt to or evade the host immune response.

Several biological studies have shown that a protein is a “social animal” [10–13], that is, a protein does not achieve its function alone but cooperates with other proteins to perform that function. Thus, most processes in a living cell are accomplished through protein-protein interaction networks; therefore, these play a central role in most activities involving the structure and function of the cell. These include signal transduction, protein folding, cell cycle control, DNA replication and transport, cellular motion, and most regulatory mechanisms [14, 15]. These interactions are of various types, but a high level description of biological systems partitions them into two categories, namely, physical and functional interactions [16]. Physical interactions refer to physical contact between proteins, and functional interactions or relationships between proteins involve the mechanism through which a particular protein achieves its functions. While “functional interactions” between proteins suggest direct physical contact between them [17], it is actually a broader concept and does not necessarily involve direct physical interactions [15].

In this work, we only refer to functional interactions, including physical and genetic interactions, and those derived from knowledge about coexpression and shared evolutionary history or pathways. Proteins interact directly or indirectly through one or more intermediates to carry out their functions in promoting the stability and robustness of the system. These interactions can be modeled as a network, called a protein-protein functional network or interactome. This is a network in which nodes or vertices are proteins and edges or links represent pairwise interactions or functional relationships between proteins within an organism. Analytically, protein-protein functional interaction networks are represented as a couple *G*(*𝒩*, *ℒ*), where *𝒩* is the set of proteins (nodes) and *ℒ* the set of functional relationships (links), and graphically visualized using an undirected graph layout representing the paths of communication and metabolism of an organism. Even though interaction networks do not directly encode cellular processes nor provide information on dynamics, they do represent a first step towards description of cellular processes, which are ultimately dynamic in nature [18], and they constitute a significant step toward understanding the functional organization of the cell [15]. Therefore, knowledge of protein-protein networks might advance our understanding of biological systems including molecular pathways and elucidate the role of various proteins in complex diseases and how they cooperate to achieve a higher goal in the host.

The most commonly used integrated functional interaction networks for many organisms [19–21] are obtained from the Search Tool for the Retrieval of Interacting Genes/Proteins (STRING) database [22, 23]. The STRING scoring system for protein or gene interactions is benchmarked by the Kyoto Encyclopedia for Genes and Genomes (KEGG) database [24] in which only 1028 out of more than 4000 encoded proteins in the MTB proteome have a known pathway, representing about 25%. This constitutes the biggest limitation for scoring newly discovered interactions between genes and/or proteins, specifically for MTB, which is not a model organism. In addition, the experimental data in the STRING database for this particular organism is limited. As an illustration, when dealing with microarray data, the STRING database retrieves its coexpression interactions from ArrayProspector (http://www.bork.embl.de/ArrayProspector) [25]. However, a large amount of microarray data for MTB are being generated and are publicly available in other resources, and these may increase the accuracy and precision of STRING data. In the case of homology data, the STRING scoring system uses the *E*-value obtained from sequence similarity searches. However, there are also protein signature databases such as InterPro [26], which is an integrated database for protein families and domains (http://www.ebi.ac.uk/interpro) [27] and can be used to increase the reliability and coverage of these homology data. Therefore, there is a need for an effective scoring system to fill gaps found in homology and microarray data in STRING for this specific organism to produce a more complete MTB functional interaction network.

To obtain a high coverage protein-protein interaction network, every functional relationship or interaction between proteins should be depicted. These interactions are discovered by various experimental approaches and often partially complemented with prediction techniques [22]. One of the subjects of heated debate around protein-protein interaction networks is that a network obtained from high-throughput experiments roughly maps the “current” network of interactions occurring inside the cell. In addition, there are several issues related to high-throughput data, including noise, environment, and the nature of the approaches used for each experiment [28]. Thus, each specific approach may incorrectly classify interactions, that is, either failing to detect interactions, referred to as false negatives or wrongly identifying some other interactions, referred to as false positives. The lack of appropriate techniques to address these shortcomings results in biases in the outputs and this is obviously a technology-dependent problem. In order to alleviate the former issue, data integration combining information from multiple interacting data sources into one unified network is deployed, leading to a higher confidence and an increased coverage. For the latter issue, a reliability threshold is applied, thus discarding all functional interactions whose reliability or confidence score is less than the threshold. These techniques are expected to significantly reduce the false negative and positive rate of the network produced, thus yielding a network of high confidence interactions. 

For the *Mycobacterium tuberculosis* (MTB) strain CDC1551, we used contributions from both primary data, such as genomic sequences and functional data from high-throughput experiments, to construct a protein-protein functional interaction network. Such a network allows us to unravel the underlying principles of its biological properties for the purpose of building a predictive disease model and identifying novel therapeutic drug targets. We performed computational analysis on the network to detect the key principles driving the biological organization of the organism and to identify proteins that are potentially indispensable for the survival and viability of the organism, referred to as *essential proteins*, and those which contribute to the fitness of the organism, referred to as *supplementary proteins*. We explored (1) the interplay between each protein pair in the network and their possible contribution to disease and (2) how they reliably function for the survival and fitness of the organism on the basis of the network topology. This categorization can provide clues toward finding effective drug targets and possibly lead to new antituberculosis compounds with novel mechanisms of action against essential proteins [29]. 

## 2. Materials and Methods

**Figure 1:**
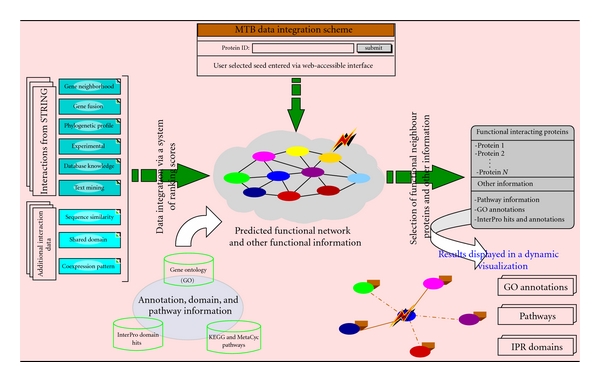
Data integration scheme.

An MTB functional interaction network was built by integrating interaction datasets from the STRING database and additional interaction data derived from sequence similarity and signature, and microarray data. The STRING database [22, 23] integrates known and predicted protein-protein associations derived from high-throughput experimental data, the mining of databases and literature, and from predictions based on genomic analysis for a large number of organisms. Functional interactions from the STRING database are used with confidence scores as defined by the STRING schemes. These include conserved genomic neighbourhood, gene fusion events, phylogenetic profile, or gene cooccurrence across multiple genomes, text mining, experiments, and other databases (http://string-db.org/). Additional interaction data are derived from protein sequence similarity and signatures, and microarray data. Functional interaction pairs predicted from protein sequence similarity and conserved protein signatures are scored using information theoretic-based approaches which translate into confidence scores for protein conserved features from evolution [30]. We used a random partial least squares regression technique for inferring genes with similar expression profiles from multiple public microarray datasets and generating functional connection scores between proteins [31]. The combined link confidence score between two proteins *i* and *j* for an integrated view of all datasets through a unified network as shown in Figure 1 is given by

(1) $$\begin{array}{c} {𝒮}_{ij}=1-\overset{9}{\underset{d=1}{\prod }}\left(1-s_{ij}^{d}\right) \end{array}$$

under the assumption of independency, and where *s*_*ij*_^*d*^ is the confidence score of a functional interaction between *i* and *j* predicted using the type of data *d*.

This section describes network centrality measures that are used to numerically characterize the importance of proteins in the system, and their contribution to the functioning of the system, thus assessing the topological significance of these proteins within the network and quantifying the structural properties of the functional network produced. These measures include degree or connectivity, betweenness, closeness, and eigenvector centrality metrics. We denote by *G*(*𝒩*, *ℒ*) the MTB functional network, with *𝒩* the set of interacting proteins and *ℒ* the set of functional interactions or connections between proteins, represented by the adjacency matrix *𝒜*, an *n* × *n* symmetric matrix, where *n* = |*𝒩*| is the number of proteins in the network and whose components *a*_*pq*_ are defined as follows:

(2) $$\begin{array}{c} a_{pq}=\{\begin{array}{cc} 1 & \text{if}\;\text{the}\;\text{protein}\;p\;\text{is}\;\text{functionally}\;\text{linked}\;\text{to}\;\\ & \text{the}\;\text{protein}\;q,\\ 0, & \text{otherwise}. \end{array} \end{array}$$

Proteins in *𝒩* are numbered from 1 to *n*, and a protein *p* is represented by its position number denoted by *p*. The adjacency matrix *𝒜* is symmetric, since if the protein *p* is functionally linked to the protein *q*, then clearly the protein *q* is also functionally linked to the protein *p*. Note that a given protein *p* is not functionally linked or connected to itself, that is, *a*_*pp*_ = 0.

*π*(*p*, *q*) denotes the distance between proteins *p* and *q* or the length of the shortest path from a protein *p* to a protein *q*, that is, the number of links in the shortest path between *p* and *q* for an unweighted graph; the shortest path between proteins being the path with the minimum number of edges connecting these proteins. If no path exists between proteins *p* and *q*, then *π*(*p*, *q*) = *∞*.

### 2.1. Degree and Betweenness Centrality Metrics

The degree or connectivity of a protein *p* is the number of links connected to it, that is, the number of its interacting neighbors [32] given by

(3) $$\begin{array}{c} \deg\left(p\right)=\underset{q\in 𝒩}{\sum }\delta \left(p,q\right), \end{array}$$

where

(4) $$\begin{array}{c} \delta \left(p,q\right)=\{\begin{array}{cc} 1 & \text{if}\;\text{the}\;\text{protein}\;q\;\text{is}\;\text{functionally}\;\text{linked}\;\text{to}\;\\ & \text{the}\;\text{protein}\;p,\\ 0 & \text{otherwise}. \end{array} \end{array}$$

In terms of the adjacency matrix *𝒜*, the degree of a protein *p* is simply the sum of components in the row or the column corresponding to the protein *p*, given by

(5) $$\begin{array}{c} \overset{n}{\underset{q=1}{\sum }}a_{pq}=\deg\left(p\right)=\overset{n}{\underset{q=1}{\sum }}a_{qp}. \end{array}$$

In fact, the degree or connectivity of a protein provides an indicator of its influence on the biological processes occurring in the organism, meaning that a protein with more functional connections tends to contribute to several processes, and may thus be a key protein in the functioning of the system.

The betweenness centrality of a protein *p* in a functional network is a metric that expresses the influence of *p* relative to other proteins within the network. It is based on the proportion of shortest paths between other proteins passing through the protein target [33] and shows the importance of a protein for the transmission of information between other proteins in the network. This metric provides an indication of the number of pairwise proteins connected indirectly by the protein target through their direct functional connections. The betweenness, *B*(*p*), of a protein *p* is given by

(6) $$\begin{array}{c} B\left(p\right)=\underset{\left(s,t\right)\in {𝒩}_{p}}{\sum }\frac{\sigma_{st}\left(p\right)}{\sigma_{st}}, \end{array}$$

where *σ*_*st*_(*p*) is the number of shortest paths from protein *s* to protein *t* passing through *p*, *σ*_*st*_ the number of shortest paths from *s* to *t* in the functional network, and *𝒩*_*p*_ = {(*s*, *t*) ∈ *𝒩* × *𝒩* : *s* ≠ *p* ≠ *t*  and  *s* ≠ *t*}. The normalized betweenness of a protein *p*, lying between 0 and 1, is given by

(7) $$\begin{array}{c} B\left(p\right)=\frac{1}{\left(n-1\right)\left(n-2\right)}\underset{\left(s,t\right)\in {𝒩}_{p}}{\sum }\frac{\sigma_{st}\left(p\right)}{\sigma_{st}}. \end{array}$$

Thus, proteins with high betweenness are expected to ensure the connectivity between proteins in the functional network and are able to bridge or disconnect connected components. As the MTB functional network generated has a scale-free property, such proteins are hubs, referring to proteins that are highly connected and serve to hold together a large number of proteins with low degree, thus integrating all proteins in a given connected component into a unified complex system. These proteins are of utmost importance for the integrity and the robustness of the system and are responsible for the small world property since connections between proteins are relatively short via these hubs.

### 2.2. Closeness and Confidence Measures of a Protein

The status, *S*(*p*), of a protein *p* in a connected network is the average distance to all other proteins, that is, the ratio of the sum of *π*(*p*, *q*) for all proteins *q* in the network to the total possible number of such paths, which is (*n* − 1). It is given by

(8) $$\begin{array}{c} S\left(p\right)=\frac{1}{\left(n-1\right)}\underset{q\in 𝒩}{\sum }\pi \left(p,q\right). \end{array}$$

The closeness measure, *𝒞*_*s*_(*p*), of a protein *p* is the inverse [32] of its status and reflects the ability of the protein to access information via other proteins and to propagate information through the network. As the MTB functional network is not completely connected, this closeness measure is calculated for each connected part separately and normalized to (*n*_*c*_ − 1)/(|*ℒ*_*c*_| − 1) [34], where *n*_*c*_ is the number of nodes in the connected part of the graph containing the node and |*ℒ*_*c*_| its size, that is, the number of functional links in the connected component. This is to make the scale uniform for comparison. Thus, the closeness measure of a protein *p* is given by

(9) $$\begin{array}{c} {𝒞}_{s}\left(p\right)=\frac{\left|{ℒ}_{c}\right|-1}{\left(n_{c}-1\right)\times S_{r}\left(p\right)} \end{array},$$

where *S*_*r*_(*p*) is the status of *p* relative to its connected component.

The closeness measure is high for a protein that is central since it has a shorter distance on average to other proteins. We define the center of gravity *𝒢*_*c*_ of the network as the set of proteins that maximize the closeness measure to any other protein in the network, given by

(10) $$\begin{array}{c} {𝒢}_{c}=\left\{p\in 𝒩:{𝒞}_{s}\left(p\right)=\underset{q\in 𝒩}{\max}\;{𝒞}_{s}\left(q\right)\right\}. \end{array}$$

The eccentricity, *E*(*p*), of a protein *p* in a given connected graph is the maximum length of shortest paths from protein *p* to all other proteins in the network, that is,

(11) $$\begin{array}{c} E\left(p\right)=\max\left\{\pi \left(p,q\right):q\in 𝒩\right\}. \end{array}$$

In the context of the MTB functional network, the eccentricity *E*(*p*) of a protein *p* is computed according to its connected component, and we consider the inverse of the eccentricity obtained, and normalize it, as done previously. The measure is referred to as the confidence *𝒞*_*e*_(*p*) of protein *p*, expressing its capability to quickly communicate with other proteins in the network, and given by

(12) $$\begin{array}{c} {𝒞}_{e}\left(p\right)=\frac{\left|{ℒ}_{c}\right|-1}{\left(n_{c}-1\right)\times E_{r}\left(p\right)}, \end{array}$$

where *E*_*r*_(*p*) is the eccentricity of *p* relative to its connected component.

The higher the confidence of a protein in the functional network, the quicker it communicates with other proteins in the network. We define the reference center *ℛ*_*c*_ of the network as the set of proteins that maximize the confidence of any other protein in the network, given by

(13) $$\begin{array}{c} {ℛ}_{c}=\left\{p\in 𝒩:{𝒞}_{e}\left(p\right)=\underset{q\in 𝒩}{\max\;}{𝒞}_{e}\left(q\right)\right\}. \end{array}$$

### 2.3. Eigenvector Centrality Metric

The degree or connectivity metric provides a simple number of functional connections without weighting them. The eigenvector metric considers the importance or weight of these functional connections [32]. In fact, functional connections are not equally important and functional connections to more influential proteins will impact more on the contribution of the protein than functional connections to less influential proteins. Thus, the eigenvector centrality metric assigns a relative weight to all proteins in the network based on the fact that functional connections to proteins of high weight contribute more to the weight of the protein target.

Let *c*_*p*_ be the numerical value representing the contribution of the protein *p* to the functioning of the system. *c*_*p*_ is then proportional to the contributions of its neighbors to the system. This means that

(14) $$\begin{array}{c} \overset{n}{\underset{q=1}{\sum }}a_{pq}c_{q}=\lambda c_{p} \end{array},$$

where *λ* is constant for every protein *p* in the functional network. In matrix form, this can be written as follows:

(15) $$\begin{array}{c} 𝒜c=\lambda c \end{array},$$

where *c* = (*c*_1_,…,*c*_*n*_)^*T*^, the transpose of the vector (*c*_1_,…, *c*_*n*_), which defines a vector of contributions of each protein. The vector *c* is an eigenvector of the adjacency matrix *𝒜* associated with eigenvalue *λ*. It is known that *λ* is the largest eigenvalue of the adjacency matrix and *c* is its nonnegative corresponding eigenvector [32, 35]. In this metric, the contribution of a given protein to the functioning of the system depends not only on the number of its interacting neighbors but also on the quality of these neighbors. Proteins with a high number of functional interactions are important, but a protein with a small number of high-quality functional connections may contribute more to the survival of the organism than one with a large number of low-quality functional connections.

## 3. Results and Discussion

We have generated an MTB functional interaction network from nine biological data sources, and the summary of number of interactions and confidence scores is shown in Table 1. For each evidence source, functional interaction scores are categorized into three different confidence levels, namely, low, medium, and high confidence. The final row shows the number of interactions in each confidence range for the final combined score. Note that for a given data source, all interactions whose scores are strictly less than 0.3 (<0.3) are considered as low confidence, scores ranging from 0.3 to 0.7 (0.3 ≤ score ≤ 0.7) are classified as medium confidence, and scores greater than 0.7 (>0.7) yield high confidence. Furthermore, the confidence increases when interaction data are integrated into a single network, producing more medium and high confidence links in the last row than when considering only one type of data. To understand the biological organization of the organism from its protein functional network and use this as a means to develop appropriate treatment strategies for the disease, complete knowledge of the network structure and the contribution of each protein to the system's biological processes are required. To this end, network centrality measures are used to reveal proteins which are potentially crucial to the functioning of the system, thus contributing to the survival of the organism.

### 3.1. General View of the MTB Functional Network

The use of these nine different biological sources is expected to solve the problem of interaction incompleteness. On the other hand, to reduce the impact of bias in functional interactions coming from experimental predictions and computational approaches, we have only considered those ranging from medium to high confidence and for functional interactions with low confidence, only those predicted by at least two different approaches were considered. In total, 5 interactions of low confidence predicted by at least two different approaches have been included in the functional network. We analyzed the network for its general properties, and these network parameters are presented in Table 2.

**Figure 2:**
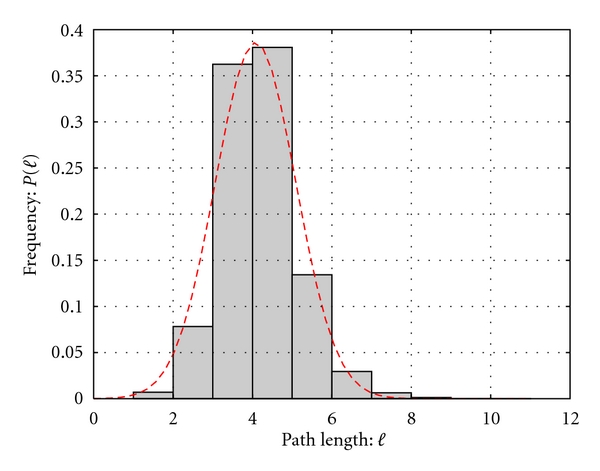
Distribution of shortest path lengths between reachable pair-wise protein functional interactions.

The network is comprised of 4136 proteins out of 4195 found in the complete list from the UniProt database [38–40], covering approximately 98.6% of the MTB proteome. Of these, 201 are structural hubs, or “single points of failure”, which are able to disconnect the network, thus affecting function, and they are considered to be responsible for the integrity of the system. Due to the presence of these hubs, any pairwise protein set in a given connected component can communicate through its relative shortest paths. In the MTB functional network, the average path length, obtained by averaging over all shortest paths between all pairs of nodes, is approximately 4 as shown in Figure 2 representing the probability distribution of the shortest path length.

 This reveals that the transmission of biological information from a given protein to others is achieved through only a few steps. Indeed, the average shortest path length value is 3.678, which is approximately of the order of magnitude log⁡(|*𝒩*|) with |*𝒩* | = 4136. This means that the MTB functional network has a “small world property” [41, 42], and the value provides an idea about the network navigability, indicating how fast the information can be spread in the system independently of the number of proteins. This property may also provide the organism with an evolutionary advantage in the sense that the system would be able to efficiently respond to perturbations in the environment and to quickly exhibit a qualitative change of behaviour in response to these perturbations.

**Figure 3:**
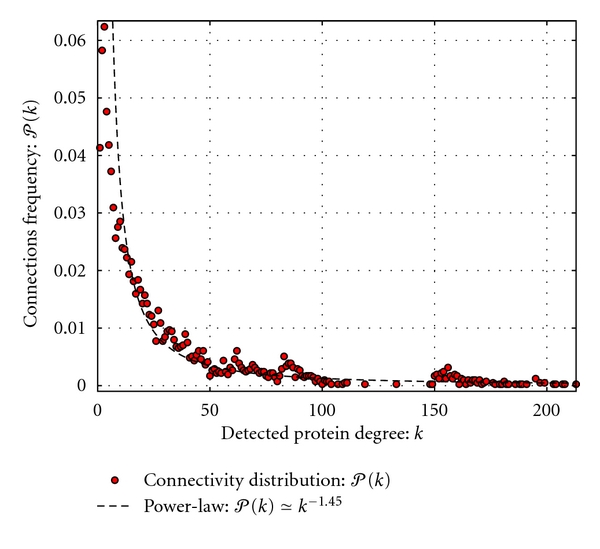
Connectivity distribution of detected *k* functional links per protein, plotted as a function of frequency *𝒫*(*k*).

 We further performed analysis of the degree distribution of the MTB functional network and, as shown in Figure 3, the functional network exhibits scale-free topology, that is, the degree distribution of proteins approximates a power law *𝒫*(*k*) = *k* ^−*γ*^, with the degree exponent *γ* ~ 1.45. This means that most of the proteins have few interacting partners but some have many partners. The latter are referred to as “high degree nodes” and probably ensure some basic chemical operations such as energy transfer and redox reactions, essential for the survival of the organism.

### 3.2. Assessing High-Degree Proteins

**Figure 4:**
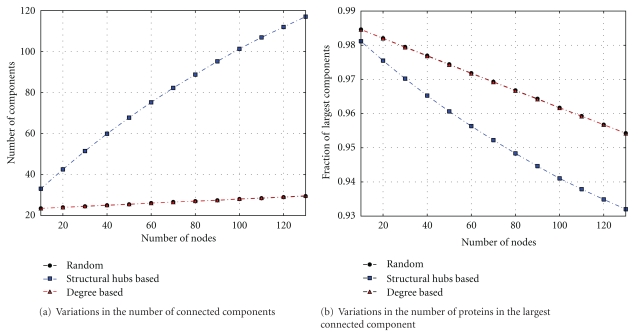
Assessing network vulnerability under random and targeted attacks.

The MTB functional network exhibits a “scale-free” property, as such it is expected to be vulnerable against targeted attack and robust against random attack. The robustness of the system is observed through its stability, expressed by its ability to remain nonvulnerable under changing environmental conditions or stressful perturbations due to a protein knockout or attack. Topologically, this can be seen as the potential connectivity of the network under a protein disruption. Thus, to assess the topological essentiality of MTB proteins, we classify them in two categories, namely, proteins with a high degree referred to as degree-based hubs and those able to disconnect the functional network, known as structural hubs. A protein is considered to be a degree-based hub if its degree is above the average degree of proteins in the MTB functional network, which is 28. We first observe the changes in the number of connected components and in the number of proteins in the largest connected component by repeatedly (1) knocking out randomly selected proteins referred to as random attacks, (2) disrupting the highest degree proteins, referred to as degree-based hub attack, and (3) removing proteins able to disconnect the network, referred to as structural hub attack. To simulate an attack, a given number of proteins are chosen for each category and the process is repeated 1000 times by randomly choosing proteins and computing the average number of the resulting components and the number of proteins in the largest component. Results are shown in Figure 4 and indicate that the MTB functional network is vulnerable to targeted structural hub attacks.

Indeed, the more structural hubs removed, the higher the number of connected components. This means that the more structural hubs are removed, the more the network is disintegrated, whereas the disruption of randomly selected proteins, or of degree-based hubs, does not perturb the general structure of the network. This means that structural hub proteins play an essential role in the network integrity. Therefore, knocking out these proteins may disturb the functioning of the system and negatively impact on the ability of this pathogenic bacterium to carry out its role in the host. We have also analyzed the network connectivity by observing the size of the largest connected component.  Figure 4(b) shows that the size of the largest component rapidly decreases when structural hubs are disrupted. This indicates that the network is disintegrated into several small connected components, thus showing the role played by the structural hubs in maintaining the network connectivity. 

### 3.3. Assessing Central Proteins

**Figure 5:**
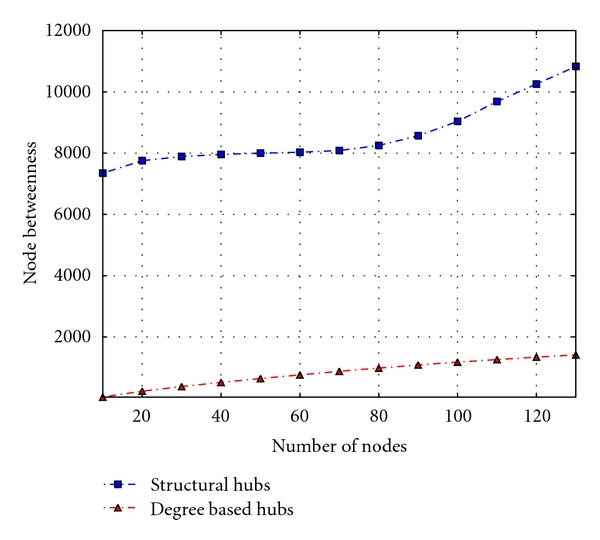
Analyzing the variations in the betweenness metric in terms of protein category.

**Figure 6:**
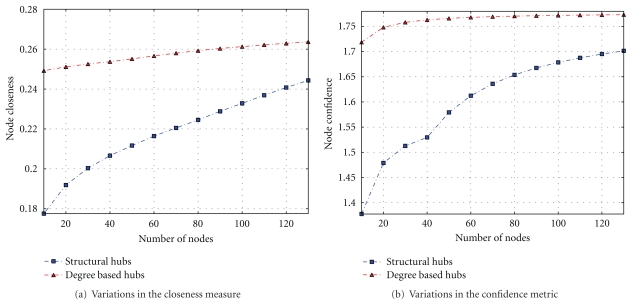
Assessing the variations in closeness and confidence centrality measures in terms of protein category.

The betweenness metric represents a significant indicator of network essentiality [42]. Proteins with high betweenness are essential to the functioning of the system, serving as bridges for communication between several other proteins in the network. A protein with high confidence or closeness will be more important because it has a smaller path length to reach all other proteins in the network, allowing the system to quickly exhibit appropriate behaviour in case of a given perturbation in the system. Figures 5 and 6 show the functional importance of proteins obtained by ordering proteins by betweenness, closeness, and confidence measures of hubs and observing the cumulative proportion for every 10 proteins. These results reveal that proteins with high degrees and located in the center of the network may reach all the nodes in a given connected component with fewer steps compared to the structural hubs. These results combined with those in Figure 6 suggest that a protein, which is a structural hub and has a high degree, is important for the survival of the bacterial pathogen. These proteins are considered to be potential drug targets and can be used to enhance the discovery process of new antibiotics with novel mechanisms of action to treat the disease.

### 3.4. Important Proteins in the MTB Functional Network

We investigated the biological significance of proteins found to be structurally important in the functional network. Specifically, we are looking at the functions that are carried out by proteins found in the center of gravity *𝒢*_*c*_ with high betweenness and connected to some influential proteins at certain levels, that is, proteins with eigenvector centrality greater than 10^−5^. We are also interested in the biological processes in which they are involved, as well as in the functional class to which they belong. This enables the identification of proteins that are potentially essential for the survival of the bacterial pathogen, as they correspond to bottlenecks in the MTB functional network and are, therefore expected to be key components of the organism's cellular processes. Bottleneck proteins are proteins responsible for several indirect functional connections between other proteins in the functional network. As the average shortest path length is 3.678, a protein in the functional network is said to belong to the gravity center if its closeness metric, as defined in (9), is greater than 1/3.678, which represents approximately 0.27189. In the case of the betweenness measure, a protein with betweenness above the total number of shortest paths expected to pass through the protein in the functional network is of interest, and this number is about 15212.21. Through these, we identified a set of 881 proteins, which constitute a set of important proteins and thus potential drug targets within the bacterial pathogen.

It is difficult to validate a set of potential drug targets computationally, and of course there are many other factors involved in determining suitability as a drug target, apart from the target having a potentially important functional role. However, we tried to assess the candidate list by looking at some known drug targets as well as targets predicted by other groups. We identified “validated” drug targets in MTB on the TDR targets website (http://tdrtargets.org). According to their documentation, these validated targets are manually curated from the literature. We also checked UniProt annotation for MTB, searching for the “Miscellaneous” comment “was identified as a high confidence drug target”, and looked at a handful of genes reported to be predicted drug targets in a table of a recent paper by Kinnings et al. [36].  Table 3 in this paper [36] lists 12 genes with a high “Target Chemical Druggability Index”. Our list of candidate drug targets includes 33 genes which were TDR validated targets, 7 of which were also in the UniProt target list, and 1 of which was also predicted by Kinnings et al. Our list included an additional 6 proteins from the Kinnings prediction, and an additional 75 proteins in the UniProt target list. Therefore, 114 proteins in our candidate list have previously been predicted or reported to be drug targets. Within this set are four known targets of existing antitubercular drugs. Two genes, inhA and folA, are known to be targets of or affected by isoniazid (inhA was also on the TDR, UniProt, and Kinnings list), embA is a known target of ethambutol, and rpoB is involved in rifampicin resistance. One protein known to be involved in activating isoniazid, KatG, was not on the list, but when we checked its network properties, its closeness and betweenness measures were only just below the cut-off for inclusion.

**Figure 7:**
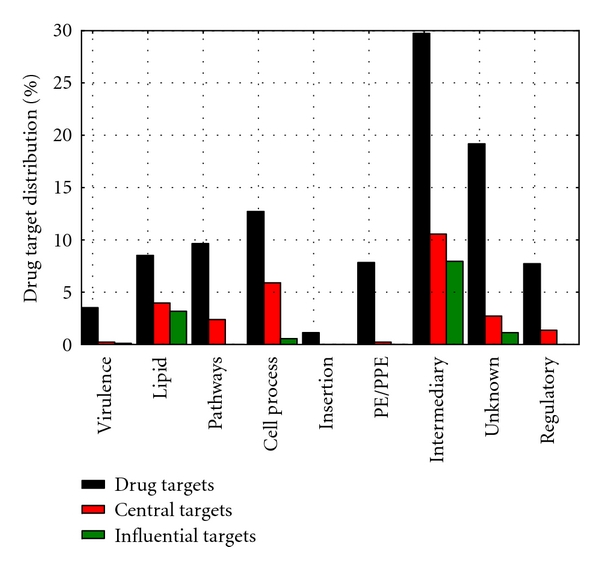
Distribution of candidate drug targets per functional class.

Further functional analysis on the candidate list was performed using a small group of high level functional classes assigned to all the proteins. These functional classes were extracted from TubercuList (http://genolist.pasteur.fr/TubercuList/), and the distribution of these potential drug targets per functional class is shown in Figure 7. These results indicate that most of the candidate drug targets are involved in intermediary metabolism, followed by a significant proportion of proteins in the unknown class and those belonging to the cell wall and cell process functional classes. We used the Fisher's Exact Test to find overrepresented functions in sets of proteins with different network properties.  Table 3 shows that the hub, high degree (50–99), and high betweenness proteins, as well as the predicted drug target list are significantly over-represented by PE/PPE proteins. Note that this distinct functional class consists of proteins whose sequences have characteristic motifs Pro-Glu at positions 8-9 and Pro-Pro-Glu at 8–10 [1], where Pro and Glu stand for Proline (P) and Glutamic (E) amino acids. Most of these proteins are specific to mycobacteria and have been suspected to allow MTB to adapt to its environment during infection or transmission [43] and to play a role in its virulence or immunogenicity [44, 45] by altering the way the host responds to the infection. Lipid metabolism proteins also feature highly in most of these lists, and regulatory proteins in some of these. One would expect regulatory proteins to be reasonably well connected as they are likely to have an effect on multiple genes. Interestingly, the high closeness measure proteins tend to be from the unknown class. The drug target list also contains 31 proteins belonging to the virulence, detoxification, and adaptation functional class. 

We also identified within the candidate drug target list, proteins which are either more central (top ranked closeness values) or more influential (top ranked eigen values) in the system and classified them per functional class, and these results are shown in Figure 7 and in Table 4. These results again show that most of the potential drug targets that are central to the functioning of the system, ensuring quick communication between proteins in the system, are involved in intermediary metabolism and respiration, cell wall and cell processes, and lipid metabolism. Some of the known antitubercular drugs target cell wall biosynthesis and lipid metabolism, in particular synthesis of mycolic acids. Those involved in intermediary metabolism and respiration, as well as lipid metabolism, are connected to proteins participating in several processes, thus playing key roles in the system. 

**Figure 8:**
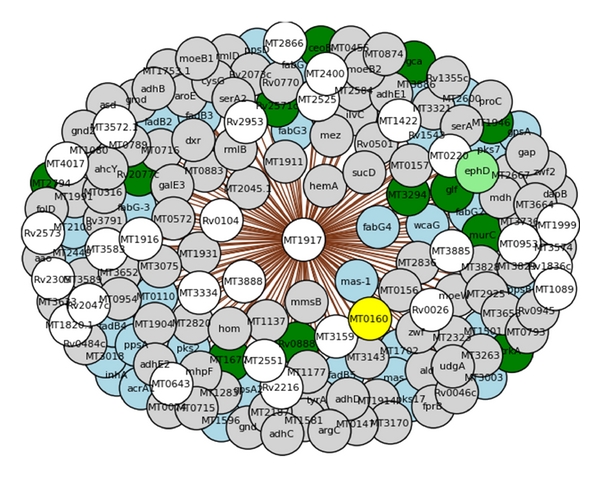
An illustration of a structural hub protein. Nodes are coloured by functional class: virulence (light-green), PE/PPE (yellow), cell wall and cell processes (green), lipid metabolism (light-blue), intermediary metabolism and respiration (grey), and unknown (white).

Many of the more influential and/or more central proteins include previously reported drug targets, for example, inhA, which is both central and influential. Therefore, these criteria could be used to rank the 114 targets. However, there are also some highly influential and central proteins in the complete candidate list that have not previously been identified or characterized. One of the predicted drug targets is the protein “MT1917” (UniProt accession P95147) shown in Figure 8. This protein is a structural hub but is uncharacterized and has been identified to be a central and influential target. It is shown to be linked to a number of proteins with various different functions and is an example of a protein that should be further investigated as a potential drug target. There are many other novel candidates with these properties, but which have also been shown to be essential for growth [37] or intracellular survival [46] that could be pursued. 

Sassetti and colleagues [37, 46] published two lists of genes from MTB H37Rv that have been shown to be involved in either normal growth or for survival during infection. These genes were mapped to CDC1551 identifiers using the orthologues file from the EBI Integr8 project [47] (http://www.ebi.ac.uk/integr8) and the network properties for these genes are summarised in Table 5. The set of genes required for normal mycobacterial growth tend to have higher average Eigenvector, betweenness, closeness, and degree values than the overall proteome. For those required for infection, these values are generally higher than the total average, but not as high as for the growth set. Of the 881 drug target proteins, 197 are on the list of proteins required for growth, and 38 are on the list of proteins required for survival during infection. This enhances their suitability as drug targets, since they have been shown experimentally to be required by the organism. 51 of the proteins required for growth and 7 proteins required during infection overlap with the 114 previously predicted drug target list mentioned above. Based on different criteria, we have ranked our complete list and show the top 10 candidates in Table 6. 

## 4. Conclusions

In this study, we have produced an MTB functional network and elucidated proteins which are essential to the functioning of the system using the network centrality measures. We showed that proteins contributing to the survival of the bacterial pathogen within the host are potential drug targets and many have previously been identified as such by different methods. These data can be used to enhance the discovery process of new drugs in order to overcome the disease caused by this particular organism, which currently constitutes a public health challenge. 

Drug targets have been traditionally identified through complete knowledge of individual proteins and their well-characterized functions. Here, we integrate biological data from different sources into a single functional network to provide a systems view of the whole bacterial pathogen for the identification of new potential drug targets. This has enabled us to identify key proteins which are still uncharacterized. It might help us to better understand the biology of the organism as a whole system and may constitute a useful tool for orienting further experiments. Furthermore, this may contribute to the process of developing new antibiotics with novel mechanisms of action for better treatment of the disease by saving time and reducing the cost. 

By combining our predicted candidate list with other drug target lists as well as gene essentiality data, we can rank the candidates according to different criteria. Some of the known targets for existing anti-tubercular drugs are not in the Sassetti et al. essential gene lists, and some of the previously reported targets are not necessarily the most central or influential; therefore, there does not appear to be a single rule for identifying the best targets. It is through integration of data that we will become better informed on target suitability. The Kinnings et al. [36] prediction uses protein 3D structure data together with drug-protein interface information, which is quite different to the approach of gene essentiality, but together these data can be used to refine drug candidate lists to find the most suitable targets. 

As the disease is a balance between virulence at the bacterial pathogen and host resistance, knocking out a given protein within the parasite may adversely impact the host system. This means that for a drug target to be effective it must take into account the host system. There is, therefore, a need to also consider the host system in order to produce a comprehensive map of protein interactions between pathogen and the human host. Thus, future plans include development of a host pathogen interaction map for MTB and human. Furthermore, since most of the hubs are of unknown function, it would be important to predict their functions, which is another direction we have pursued.

##  Authors' Contribution

N. J. Mulder generated and supervised the project, and finalized the manuscript. G. K. Mazandu analyzed, designed and implemented the model, and wrote the manuscript. N. J. Mulder and G. K. Mazandu analyzed data, read, approved the final manuscript and N. J. Mulder approved the production of this paper.

##  Conflict of Interests

The authors declare that they have no conflict of interests. 

## Figures and Tables

**Table 1:** The number of associations in the MTB functional network, shown separately for each data source and confidence range from low to high.

Association evidence by type	Low confidence	Medium confidence	High confidence
Conserved genomic neighbourhood	1163	6972	4731
Gene fusion events	337	52	99
Phylogenetic Profile	1033	5862	1461
Text mining	1174	722	93
Experimental	220	170	133
Knowledge from database	3	970	2002
Sequence similarity	8524	1345	77
Shared domains	0	20915	17792
Coexpression	6538	225	4

Combined score	6850	32488	25605

**Table 2:** General MTB functional network parameters.

Parameters	Value
Number of proteins (Nodes)	4136
Number of functional interactions (Edges)	58098
Average degree (in and out)	28
Average shortest path length	3.678
Number of connected components	23
% of Nodes in largest component	98.7%
Number of hubs	201

**Table 3:** Summary of overrepresentation analysis of functional classes for different protein sets based on network properties.

Protein set	Overrepresented function	*P* value	Adjusted *P* value
Hubs	PE/PPE	2.10576*e* − 05	1.89518*e* − 04

Degree ≥ 100	Lipid metabolism	4.37537*e* − 12	1.96891*e* − 11

	Intermediary metabolism and respiration	1.06668*e* − 25	9.60013*e* − 25
	Lipid metabolism	2.33426*e* − 08	7.00278*e* − 08
Degree 50–99	Information pathways	0.0209259	0.0470832
	Regulatory proteins	1.91556*e* − 52	8.62003*e* − 52
	PE/PPE	8.196*e* − 115	7.3764*e* − 114

	Lipid metabolism	0.00358115	0.0080576
	Intermediary metabolism and respiration	1.33874*e* − 58	1.20487*e* − 57
Degree 10–49	Information pathways	2.6561*e* − 10	7.96829*e* − 10
	Virulence, detoxification, adaptation	4.90211*e* − 11	2.20595*e* − 10

	Unknown	4.98171*e* − 180	4.48354*e* − 179
Degree <10	Cell wall and cell processes	4.47945*e* − 04	1.78646*e* − 03
	Insertion seqs and phages	5.95487*e* − 04	1.78646*e* − 03

	Lipid metabolism	2.03723*e* − 04	3.66702*e* − 04
	Intermediary metabolism and respiration	5.99428*e* − 08	1.79828*e* − 07
Betweenness >15 000	Information pathways	1.54837*e* − 06	3.48383*e* − 06
	Regulatory proteins	3.51658*e* − 08	1.58246*e* − 07
	PE/PPE	4.48875*e* − 11	4.03987*e* − 10

Closeness >0.5	Unknown	2.58864*e* − 14	2.32978*e* − 13

Eigenvector >0.08	Lipid metabolism	1.5511*e* − 12	6.97994*e* − 12
	Intermediary metabolism and respiration	2.85447*e* − 31	2.56902*e* − 30

	Lipid metabolism	2.68651*e* − 05	4.83571*e* − 5
	Intermediary metabolism and respiration	2.12524*e* − 11	9.56358*e* − 11
Drug target	Information pathways	4.13904*e* − 07	9.31285*e* − 07
	Regulatory proteins	6.36758*e* − 08	1.91027*e* − 07
	PE/PPE	1.53973*e* − 12	1.38576*e* − 11

**Table 4:** Repartition per class of potential drug target proteins, considering those which are central and those considered to be more influential.

	Functional class	Proteins	Drug targets	Central targets	Influential targets
1	Virulence, detoxification, adaptation	176	31	2	1
2	Lipid metabolism	230	75	35	28
3	Information pathways	245	85	21	—
4	Cell wall and cell processes	618	112	52	5
5	Insertion seqs and phages	82	10	—	—
6	PE/PPE	147	69	2	—
7	Intermediary metabolism and respiration	884	262	93	70
8	Unknown	1637	169	24	10
9	Regulatory proteins	176	68	12	—

	Total	4195	881	241	114

**Table 5:** Summary of network properties of protein sets from the total proteome in the network, those required for normal growth and those required for survival during infection.

Metric	Total	Growth	Survival
Average eigenvector	0.003403	0.004342	0.003486
Average betweenness	10792.87	16108.18	11487.32
Average closeness	0.28629	0.298827	0.287806
Average degree	28.082	36.95911	33.17778
% Hubs	4.859768	0.851789	3.888889

**Table 6:** Top 10 drug target candidates ranked based on criteria such as high eigenvector (influential) and closeness (central) values, previous identification as a possible target, and essentiality.

UniProt Acc	Gene name	Functional class	Network centrality scores			Previous identification sources				
			Eigen	Betweenness	Closeness	Degree	TDR	UniProt	Drugome [36]	Essential [37]
Q7D6Z3	MT2600	Lipid metabolism	0.08369	128207.32	0.37	207	Yes			Yes
P0A5Y6	inhA	Lipid metabolism	0.08216	27379.65	0.36	162	Yes	Yes	Yes	
O06934	glf	Cell wall and cell processes	0.08152	67825.15	0.36	172	Yes			
Q11141	proC	Intermediary metabolism and respiration	0.08123	57376.99	0.36	165			Yes	Yes
P63562	argC	Intermediary metabolism and respiration	0.08119	51684.48	0.36	163		Yes		Yes
P63629	hom	Intermediary metabolism and respiration	0.08137	50079.08	0.36	171		Yes		Yes
P64328	hemA	Intermediary metabolism and respiration	0.08126	77831.05	0.36	173		Yes		Yes
P0A544	serA	Intermediary metabolism and respiration	0.08133	28205.61	0.36	164		Yes		Yes
P66783	Rv3791	Intermediary metabolism and respiration	0.08162	27347.66	0.35	157		Yes		Yes
O33290	ftsK	Cell wall and cell processes	0.00172	62658.74	0.35	97		Yes		Yes
